# Extensive Odontogenic Deep Neck Infection Complicated by Necrotizing Fasciitis and Bilateral Mastitis: A Rare Case Report

**DOI:** 10.1155/cris/5614042

**Published:** 2026-04-22

**Authors:** Adil Aytaç, Uğur Dönmez, Bahar Yanık Keyik

**Affiliations:** ^1^ Department of Radiology, Balıkesir University Faculty of Medicine, Balıkesir, Türkiye, balikesir.edu.tr

**Keywords:** deep neck infection, mastitis, necrotizing fasciitis, odontogenic infections

## Abstract

**Objectives:**

Odontogenic infections are a leading cause of deep neck space infections and may progress rapidly through fascial planes, leading to severe complications such as necrotizing fasciitis, mediastinitis, and sepsis. This case represents an exceptionally rare example of odontogenic deep neck infection complicated by necrotizing fasciitis with bilateral breast involvement, emphasizing its potential for aggressive, life‐threatening spread.

**Case report:**

We describe a 58‐year‐old woman with diabetes, hypertension, and coronary artery disease who presented with neck and chest wall swelling, erythema, crepitus, and sepsis after 2 weeks of inadequate treatment for a buccal abscess. Imaging revealed multicompartment spread with subcutaneous emphysema, myositis, abscess formation, and bilateral breast involvement. The patient underwent aggressive resuscitation, broad‐spectrum intravenous antibiotics, and serial surgical debridements through extended cervical and anterior chest wall incisions, followed by negative pressure wound therapy (NPWT), resulting in clinical and laboratory improvement.

**Discussion:**

This case highlights the potential for odontogenic infections to progress beyond the neck into the thoracic wall and breast tissue, emphasizing the importance of early imaging and comprehensive source control. Diabetes was likely a significant predisposing factor for the rapid and extensive spread observed. Contrast‐enhanced CT is critical for mapping disease extent and guiding timely surgical intervention, which is associated with reduced morbidity and mortality.

**Conclusions:**

Early recognition, imaging, and a multidisciplinary treatment strategy, including prompt broad‐spectrum antibiotic therapy and repeated surgical debridement, are essential to prevent life‐threatening complications of odontogenic deep neck infections. Clinicians should maintain a high index of suspicion in patients with systemic symptoms and comorbidities, even when initial dental infections appear minor.

## 1. Introduction

Odontogenic infections arise from the dental pulp or periodontal tissues and most often present as periapical or periodontal abscesses [1]. They represent the leading cause of infections involving the oral cavity and account for ~40%–60% of deep neck infections [2, 3]. Because the clinical presentation of odontogenic infections is frequently nonspecific in the early stages, diagnostic delays are common [4]. Contributing factors include patients’ tendency to underestimate dental infections as minor problems, the masking of symptoms by early analgesic or inappropriate antibiotic use, and delayed referral to dental or surgical care [4, 5].

Although oral cavity infections typically begin as localized abscesses, they may rapidly spread through adjacent soft tissues and cervical fascial planes [6]. This progression may manifest as cellulitis and extend into deep neck spaces, leading to abscess formation, airway compromise, mediastinal spread, and sepsis [7, 8]. In severe cases, infection may progress to necrotizing fasciitis, characterized by rapid fascial spread, tissue necrosis, and systemic toxicity [9]. Extension into cervical compartments may result in mediastinitis with high mortality [10]. Secondary mastitis arising from the descending spread of a deep neck infection is an exceptionally rare clinical entity. A focused PubMed search identified only three previously reported cases of odontogenic‐origin deep neck infection extending to the mammary region in the English‐language literature. None of these cases described bilateral breast involvement [11–13]. Therefore, early recognition and aggressive treatment are essential to prevent life‐threatening complications [5, 14]. Delayed or inadequate treatment significantly increases morbidity and mortality, particularly when infection progresses to deep neck involvement or necrotizing fasciitis [11, 15]. Although odontogenic deep neck infections are relatively common, cases complicated by myositis, necrotizing fasciitis, and mastitis remain exceedingly rare [12]. Here, we present a case that began as a simple dental infection, was initially managed by a non‐specialist, and subsequently progressed to neck abscess, cellulitis, myositis, deep neck infection, necrotizing fasciitis, and mastitis. This case highlights how seemingly minor odontogenic infections can culminate in rare but life‐threatening complications [11, 12].

## 2. Case Presentation

A 58‐year‐old woman presented to the emergency department with confusion, malaise, swelling, and erythema of the neck and chest. Her past medical history was notable for coronary artery disease, diabetes mellitus, and hypertension. She reported no history of smoking or alcohol consumption, and there was no significant family history. Approximately 2 weeks earlier, she had developed swelling around a lower right tooth and sought care at an outside hospital, where a buccal abscess was diagnosed. She was hospitalized for 2 weeks and underwent an incision over the neck and chest performed by a general surgeon. Wound culture grew *Streptococcus alactolyticus*, and intravenous ceftriaxone, piperacillin‐tazobactam, and teicoplanin were initiated.

Despite treatment, the patient’s condition progressively deteriorated, prompting her to leave the outside facility and present to our emergency department. On examination, she had swelling, erythema, and warmth over the right side of the face and neck, as well as fistulous openings with purulent drainage over the neck and both breasts. Palpation revealed crepitus involving both the cervical and breast tissues. Her temperature was 39.2°C, heart rate 110 bpm, and blood pressure 85/45 mmHg. Laboratory studies showed leukocytosis (14,700/mm^3^), elevated CRP (130 mg/L), markedly increased procalcitonin (12 ng/mL), ESR (80 mm/h), and hyperglycemia (286 mg/dL).

**Figure 1:**
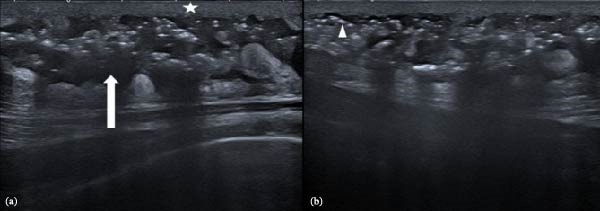
Neck ultrasonography images of a 58‐year‐old patient with deep neck infection. (a) The asterisk indicates diffuse skin thickening, and the white arrow demonstrates reticular subcutaneous edema. (b) The arrowhead highlights one of the multiple hyperechoic foci with comet‐tail artifacts, consistent with subcutaneous gas.

**Figure 2:**
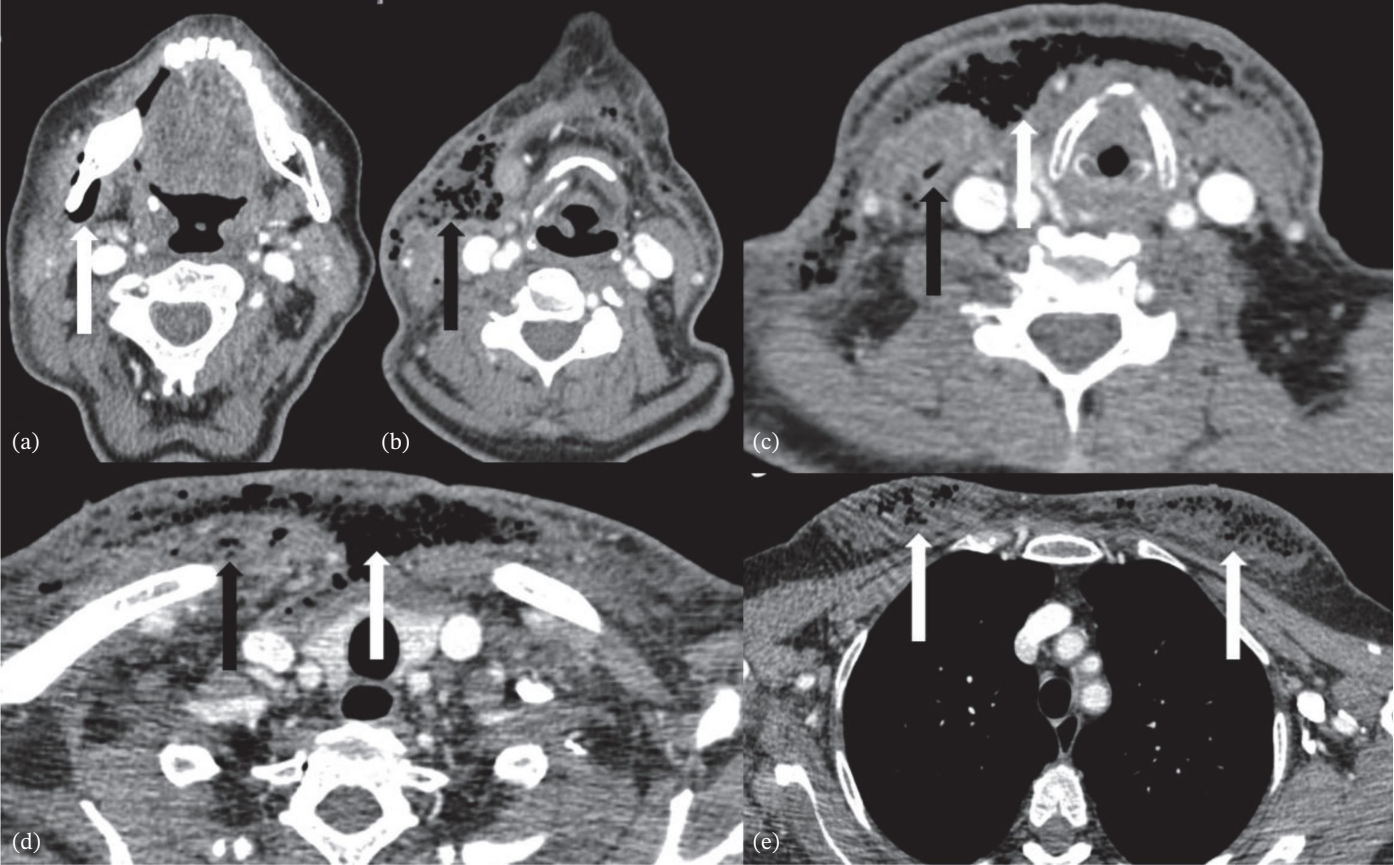
Contrast‐enhanced neck CT images of a 58‐year‐old patient with deep neck infection. (a) The white arrow indicates subcutaneous emphysema adjacent to the right mandibular ramus and submandibular gland. (b) The black arrow highlights a collection anterior to the right sternocleidomastoid (SCM) muscle, containing air foci and consistent with abscess formation. (c) The black arrow indicates intramuscular air foci within the right SCM muscle, suggestive of myositis, while the white arrow shows diffuse midline cervical emphysema. (d) The black arrow demonstrates intramuscular air within the right pectoralis major muscle, consistent with myositis, and the white arrow illustrates extensive emphysema extending from the cervical midline to the anterior chest wall. (e) The white arrow highlights intramammary air foci, compatible with mastitis.

Neck ultrasonography revealed skin thickening, increased echogenicity, reticular subcutaneous edema, and multiple hyperechoic foci consistent with subcutaneous gas (Figure 1). Contrast‐enhanced CT of the neck demonstrated extensive subcutaneous emphysema and phlegmonous soft‐tissue infiltration extending from the right mandibular ramus and submandibular gland region through the right sternocleidomastoid (SCM) muscle, superficial cervical fascia, anterior strap muscles, visceral compartment, contralateral strap muscles, bilateral pectoral muscles, anterior chest wall, and into both breasts (Figure 2). The right SCM and right pectoral muscles were thickened and edematous, with intramuscular gas. A 22 mm × 48 mm × 71 mm peripherally enhancing, gas‐containing collection was also noted in level 5b–5c (Figure 2).

The patient was diagnosed with a multicompartment, odontogenic deep neck infection complicated by abscess, cellulitis, myositis, necrotizing fasciitis, and mastitis. She was admitted to the otolaryngology service, where she received hemodynamic and respiratory support, fluid resuscitation, oxygen therapy, glycemic control, and close monitoring of organ function. Upon admission to our institution, repeat wound and peripheral blood cultures were obtained prior to modification of antimicrobial therapy. At the referring hospital, wound cultures had yielded *S. alactolyticus*. Given the patient’s septic presentation, extensive cervicothoracic necrotizing infection, and underlying diabetes mellitus, empirical broad‐spectrum intravenous antibiotic therapy was initiated in accordance with established recommendations for necrotizing soft tissue infections. Empirical treatment consisted of piperacillin–tazobactam (4.5 g every 6 h) for broad gram‐negative and anaerobic coverage, clindamycin (900 mg every 8 h) for toxin suppression in suspected streptococcal or toxin‐mediated necrotizing infection, and teicoplanin (loading dose 6–12 mg/kg every 12 h for three doses, followed by 6–12 mg/kg once daily, adjusted for renal function) to ensure adequate methicillin‐resistant *Staphylococcus aureus* (MRSA) coverage. Repeat wound cultures obtained at our institution demonstrated a polymicrobial infection, including MRSA and mixed aerobic–anaerobic flora consistent with an odontogenic origin. Blood cultures remained negative throughout hospitalization. Following antimicrobial susceptibility testing and infectious disease consultation, antibiotic therapy was tailored accordingly. Teicoplanin was continued to maintain MRSA coverage, and piperacillin–tazobactam was maintained to address Gram‐negative and anaerobic organisms identified in the polymicrobial cultures. Clindamycin was discontinued after clinical stabilization and adequate surgical source control. As inflammatory markers decreased and wound findings improved following repeated surgical debridements, antimicrobial therapy was gradually streamlined in a stepwise manner. Broad‐spectrum therapy was de‐escalated to targeted intravenous beta‐lactam–based treatment guided by susceptibility results and clinical response. The total duration of intravenous antimicrobial therapy was determined based on clinical resolution, laboratory normalization, and adequate source control. Surgical management included urgent wide cervical and anterior chest wall incisions. Cervical access was achieved through incisions involving the right submandibular and lateral cervical regions, extending along the anterior border of the SCM muscle, with additional communicating incisions across the anterior cervical compartment to facilitate adequate drainage of deep neck spaces. These were extended onto the anterior chest wall with bilateral incisions over the pectoralis major and mammary regions, allowing thorough drainage of purulent collections and complete removal of necrotic tissue across all involved compartments. This was followed by staged serial debridements of necrotic subcutaneous tissue, fascia, involved muscle, and affected breast tissue. In total, eight staged debridement procedures were performed during hospitalization. Early re‐exploration was undertaken within 24–48 h, followed by additional procedures at approximately days 3–4 and 5–7 due to ongoing necrosis. Further debridements were required between days 8–12 and 14–21, with later procedures (approximately days 21–30 and 30–40) performed to ensure complete source control and optimize wound bed preparation. Debridement was continued at each session until viable, bleeding tissue margins were achieved. Wounds were managed in an open fashion during the acute phase to allow serial reassessment. During the intermediate phase, negative pressure wound therapy (NPWT) was applied using a vacuum‐assisted closure system with reticulated open‐cell foam dressings connected to a controlled subatmospheric pressure device. This approach facilitated continuous exudate removal, reduction of tissue edema, and promotion of granulation tissue formation, thereby optimizing the wound bed prior to delayed closure. Definitive wound closure was delayed until systemic stabilization and adequate local infection control were confirmed. After approximately 6 weeks of multidisciplinary inpatient management, the patient was discharged in stable condition.

## 3. Discussion

Deep neck infections of odontogenic origin may rapidly spread through the natural anatomical pathways of the cervical fascial planes, potentially involving the mediastinum and resulting in life‐threatening complications [6, 10]. This spread is facilitated by impaired tissue perfusion, microthrombosis, and toxin‐mediated immune dysfunction [16]. Necrotizing fasciitis represents the most severe form of this process, characterized by rapid progression, gas formation, and extensive tissue necrosis [9, 15]. Reported mortality rates range from 20% to 40%, with comorbidities—particularly diabetes—exacerbating disease severity [15, 16]. In our case, the patient’s diabetes was considered a significant predisposing factor for the unusually aggressive spread of infection.

Imaging plays a pivotal role in the diagnosis of odontogenic deep neck infections, complementing clinical assessment and guiding surgical planning [6]. While ultrasound can demonstrate subcutaneous edema, hyperemia, and the presence of gas, it is limited for evaluating deep compartment spread [7]. Contrast‐enhanced CT remains the gold standard, accurately delineating gas distribution along fascial planes, fluid collections, muscular involvement, and inflammatory changes [6, 8]. Diagnostic CT findings of necrotizing fasciitis include linear or patchy gas, fascial thickening, and diffuse soft‐tissue enhancement [9, 13]. Differential diagnoses include cellulitis, isolated abscess, pyomyositis, hematoma, and traumatic edema; however, the presence of gas and rapid clinical deterioration strongly suggests necrotizing fasciitis [9]. Early CT not only improves diagnostic accuracy but also helps delineate the extent of surgical debridement required, thereby reducing mortality [5, 13].

Reports indexed in PubMed up to 2025 indicate that odontogenic necrotizing fasciitis, although rare, is associated with high mortality [11]. Gore et al. [11] conducted a systematic review of 164 patients and identified diabetes as an independent risk factor for mortality. Huff et al. [13] reported a case of mandibular infection extending to the anterior chest wall, in which survival was achieved through aggressive surgical management. Wahbi et al. [12] presented two cases in which early surgery combined with broad‐spectrum antibiotics reduced complication rates. Only a very limited number of published reports have described odontogenic necrotizing infections with cervicothoracic extension reaching the anterior chest wall and/or breast region [12, 13]. Wahbi et al. [12] reported two cases of odontogenic necrotizing fasciitis with chest wall involvement, illustrating the potential for descending spread beyond the neck compartments. Huff et al. [13] similarly described extensive cervicothoracic extension of odontogenic origin requiring aggressive staged surgical management. In contrast to these previously reported presentations, our case demonstrated exceptionally extensive multicompartment spread from the mandibular region through bilateral cervical fascial planes into both pectoral compartments and bilateral breast tissue, manifesting as secondary mastitis. This symmetric mammary involvement, together with deep cervical and muscular extension, supports the originality of the present report. Beyond anatomical extent, the therapeutic course in our case also differed in magnitude and wound‐management strategy. While prior reports emphasize early aggressive source control and broad‐spectrum antibiotics [11–13], our patient required eight staged debridement procedures over a prolonged hospitalization, reflecting the severity of necrotizing involvement and the need for repeated reassessment of tissue viability. In addition, repeat cultures after transfer demonstrated polymicrobial infection, including MRSA, necessitating combined MRSA‐directed therapy and broad gram‐negative/anaerobic coverage before stepwise de‐escalation. The use of NPWT during the intermediate phase and delayed definitive closure further highlights the staged reconstructive approach required in this unusually extensive presentation.

The combination of cellulitis, abscess, pyomyositis, necrotizing fasciitis, and mastitis—extending bilaterally to the breast tissue—distinguishes this case from most published reports, in which infections are typically confined to the mandible, submandibular region, or unilateral neck spaces [12]. This case underscores that odontogenic infections may extend beyond a localized process and result in systemic, life‐threatening complications [6, 12].

At the outside hospital, only incision and drainage were performed, which failed to achieve adequate source control and permitted further disease progression. Literature emphasizes that insufficient surgical intervention, delayed antibiotic initiation, and inadequate source control are significantly associated with mortality [15, 16]. In patients with comorbidities, early contrast‐enhanced CT, comprehensive assessment of disease extent, and prompt initiation of a multidisciplinary treatment approach are essential to reduce morbidity and mortality [5, 13].

Our management involved early empirical broad‐spectrum antibiotics, subsequently tailored to culture sensitivity results, hemodynamic stabilization, glycemic control, and serial surgical debridements. Complete removal of necrotic tissue and repeated drainage procedures were crucial in reducing the infectious burden, leading to rapid laboratory improvement [17]. Literature supports that combined surgical and medical therapy significantly improves survival and decreases recurrence risk [15, 16].

The main limitation is the lack of intraoperative and clinical photographs, which would have provided direct visualization of the necrosis extent and surgical approach.

This case demonstrates that neglected odontogenic infections can rapidly spread into deep neck spaces, anterior chest wall, and breast tissue, resulting in life‐threatening complications [6, 12]. Early CT assessment of disease extent, prompt initiation of broad‐spectrum antibiotics, timely source control, and coordinated multidisciplinary surgical management are essential to improving patient outcomes [5, 13]. Increased awareness among dentists, emergency physicians, and surgeons may facilitate earlier recognition and prompt aggressive management, ultimately improving patient outcomes [5, 17]. This case underscores the need for heightened vigilance and aggressive multidisciplinary management in odontogenic infections, particularly in patients with systemic comorbidities.

## Funding

The authors did not receive any specific grant from funding agencies in the public, commercial, or not‐for‐profit sectors.

## Ethics Statement

All procedures followed were in accordance with the ethical standards of the responsible committee on human experimentation (institutional and national) and with the Helsinki Declaration of 1975 as revised in 2008. This article does not contain any studies with human or animal subjects performed by any of the authors. Institutional review board approval was waived as only a single anonymized case is presented.

## Consent

Informed consent was obtained from all individual participants included in the study.

## Conflicts of Interest

The authors declare no conflicts of interest.

## Data Availability

The data that support the findings of this study are available upon request from the corresponding author. The data are not publicly available due to privacy or ethical restrictions.
